# Cytotoxicity of L-asparaginase from eucaryotic *Cladosporium* species against breast and colon cancer in vitro

**DOI:** 10.1186/s43046-025-00270-6

**Published:** 2025-05-03

**Authors:** Dina Johar, Hamido M. Hefny, Moselhy S. Mansy, Amal A. I. Mekawey, Mohammed S. Abdulrahman, Samy Zaky

**Affiliations:** 1 Biochemistry and Nutrition Department, Ain Shams University, Faculty of Women for Arts, Sciences and Education, Heliopolis, Cairo, Egypt; 2 Department of Microbiology and Immunology, Al-Azhar University, Cairo, Egypt; 3 Regional Center for Mycology and Biotechnology RCMB, Al-Azhar University, Cairo, Egypt; 4 Hepatogastroenterology Department, Faculty of Medicine, Al-Azhar University, Cairo, Egypt

**Keywords:** Breast cancer, *Cladosporium*, Colon cancer, L-asparaginase, MCF-7, MDA-MB-231

## Abstract

**Background:**

Recent statistical analyses indicate a rapid increase in the incidence of breast and colon cancer in Egypt. Although invasive techniques have been widely employed for early detection, diagnosis, and intervention of those cancers, they are associated with inherent risks and limitations, which often result in various complications. Therefore, noninvasive screening methods are inevitable due to their accessibility, cost-effectiveness, and high patient compliance rates. The enzyme L-asparaginase catalyzes the conversion of L-asparagine to L-aspartic acid: key metabolite for tumor cell division, thereby demonstrating anticancer potential. However, the prolonged use of bacterial L-asparaginase may cause allergic reactions and side effects such as diabetes, leukopenia, and co-agglutination disorders. Exploring the anticancer properties of L-asparaginase from different species such as yeast and fungi has been proposed to mitigate these adverse effects.

**Objectives:**

This study aimed at extracting and optimizing the expression of L-asparaginase from the eukaryotic *Cladosporium* species, as to assess its anticancer potential against breast and colon cancer cell lines.

**Method:**

*Cladosporium* species were identified morphologically and then cultured on modified Czapek-Dox Agar (mCDA) medium supplemented with L-asparagine to induce L-asparaginase production. Submerged fermentation was employed to optimize enzyme production. The enzyme activity was quantified using the Nesslerization method, and its cytotoxicity against colon and breast cancer cell lines was assessed using the (MTT) assay.

**Results:**

Among the *Cladosporium* isolates, 18.4% exhibited positive plate assay test, with enzyme activities ranging from 255 to 428 U/mL. Immunoblotting using sodium dodecyl sulfate–polyacrylamide gel electrophoresis (SDS-PAGE) analysis revealed single protein band of approximately 37 kDa, consistent with L-asparaginase activity. Cytotoxicity assay of purified L-asparaginase showed significant antiproliferative effects against breast cancer cell lines MCF-7 and MDA-MB-231, with IC_50_ values of 36.26 and 45.7 µg/mL, respectively.

**Conclusion:**

Certain eukaryotic *Cladosporium* strains are potential sources for the anticancer L-asparaginase production.

**Graphical Abstract:**

Microbial L-asparaginase is one of the most important industrial enzymes of interest accounting for about 40 % of the total worldwide enzyme sales, this enzyme has got much significance in the medical field for the treatment of leukemia especially acute lymphoblastic leukemia (ALL).

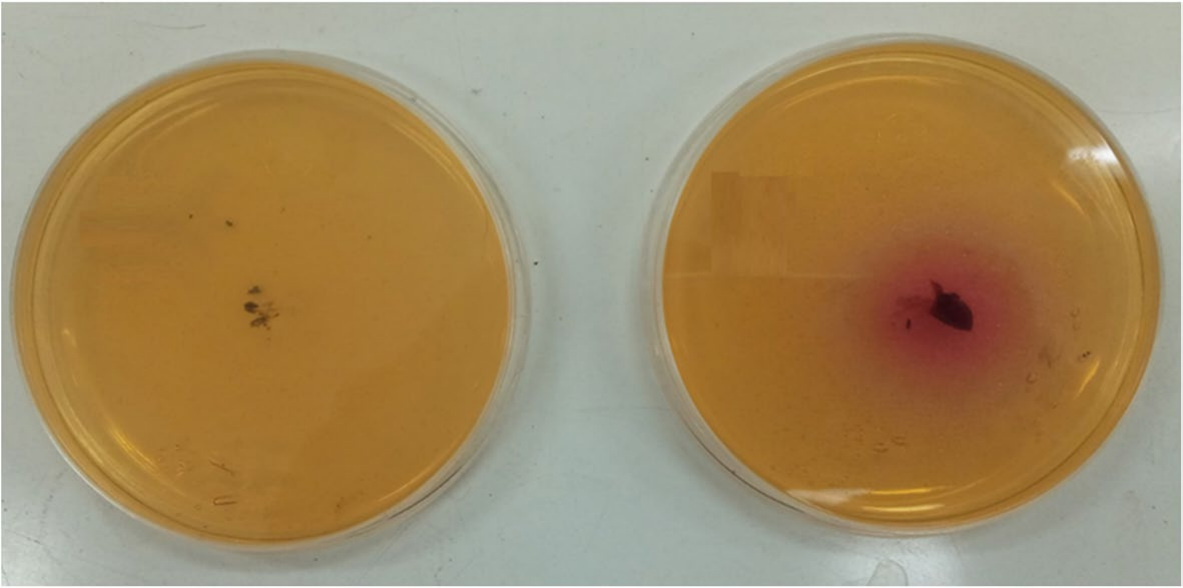

**Supplementary Information:**

The online version contains supplementary material available at 10.1186/s43046-025-00270-6.

## Introduction

In Egypt, according to the National Cancer Institute statistics, colorectal cancer (CRC) ranks the sixth most common cancer in males [1, 2], whereas breast cancer ranks the first most common cancer in females worldwide [3]. Approximately, 46,000 incident breast cancer cases are expected in 2050 [4]. Although colonoscopy is gold standard for early detection of CRC and its precancerous lesions, it has its risks and limitations and is associated with several complications [5]. Thus, noninvasive screening is of utmost importance as it is widely available, cost-effective, and has a good patient compliance [6]. Currently, the primary treatments for breast cancer are surgery and chemoradiotherapy. The development of novel therapeutics that preferentially sensitize breast and colon tumor cells to chemoradiotherapy, mitigate side effects, and improve both outcome and morbidity remains a significant challenge. *Cladosporium*, a ubiquitous fungal genus, is known for its ecological adaptability and production of bioactive compounds and enzymes with industrial and medical significance. It synthesizes metabolites like cladosporin, which possess antimicrobial, anticancer, and antioxidant properties [7]. The genus is also a source of extracellular enzymes, including cellulases, proteases, and L-asparaginase, the latter being crucial in leukemia treatment due to its asparagine-depleting capability [8]. Fungal L-asparaginase, including from *Cladosporium*, offers advantages over bacterial versions, such as reduced immunogenicity and enhanced stability [9]. Recent studies have optimized enzyme production through strain improvement and fermentation techniques, underscoring its biotechnological potential [10]. L-asparaginase catalyzes the hydrolysis of L-asparagine: an essential amino acid for tumor cell division to L-aspartic acid. Deficiency of L-asparagine can deplete tumor cells of this essential metabolite, leading to cell death. While L-asparaginase has been successfully used in the treatment of acute lymphoblastic leukemia (ALL), its potential as a therapeutic agent for solid tumors, including breast and colon cancer, is still under investigation [11]. How L-asparaginase regulates tumor progression and desensitizes cells to die is not well understood. This enhances our rationale for exploring the efficacy for targeting L-asparaginase as an approach of improved cancer therapies. Versatile microorganisms like bacteria and fungi are important sources of L-asparaginase enzyme [12]. Bacterial L-asparaginase may cause some allergic reactions and side effects like diabetes, leukopenia, and co-agglutination abnormalities in the long-term use. Exploring the anticancer effects of L-asparaginase from different species such as yeast and fungi was proposed to reduce the adverse effects [13]. The objective of this study was to optimize the production and purification of L-asparaginase enzyme from *Cladosporium* species and to assess its cytotoxicity against colon and breast cancer cells in vitro.

## Materials and methods

### Isolation and identification of *Cladosporium* isolates

Various culture media were utilized for collection, isolation, purification, and preservation of fungal isolates including dichloran glycerol agar (DGA) (Merck), potato dextrose agar (PDA) (Oxoid), Sabouraud Dextrose Agar (SDA) (HiMedia), and Malt Extract Agar (MEA) (Oxoid). Agar plates contained chloramphenicol 100 mg/L and gentamicin 40 mg/L [14]. The fungal isolates were incubated for 4–14 days at 30 °C [15, 16]. Then the 212 *Cladosporium* isolates were identified based on culture characterization, macroscopic and microscopic properties described in [17], and the biochemical reactions such as casein hydrolysis test, starch hydrolysis test, gelatin hydrolysis test, tyrosine hydrolysis test, and urease test described in [18].

### Detection of L-asparaginase enzyme from *Cladosporium* species

A mCDA medium was used for fungal isolates, supplemented with phenol red dye (2.5% in ethanol, the pH was adjusted to 7.0). The fungal isolates at a density of 1 × 10^6^ colony-forming unit/mL were inoculated and incubated at 30 °C for 48 h. The production of L-asparaginase was indicated by observing the pink zone around the colonies that was subsequently selected for the determination of enzyme activity [19].

### Qualitative assay of L-asparaginase enzyme from *Cladosporium* species

A submerged fermentation technique was used to produce L-asparaginase from *Cladosporium* isolates. In this technique, the mCDA medium contained 6.0 g of Na_2_HPO_4_.2H_2_O, 3.0 g of KH_2_PO_4_, 0.5 g of NaCl, 10-g L-asparaginase, 1M solution of 1 MgSO_4_.7H_2_O (2.0 mL), and 0.1M solution of CaCl_2_.2H_2_O (1.0 mL), 20% glucose stock (10.0 mL), and 20.0 g of agar per liter of distilled water. The pH was adjusted to 6.2, and the broth was then autoclaved [16]. At the end of the incubation period (7 days), culture filtrates were centrifuged at 7000 rpm for 15 min to obtain the supernatant using Eppendorf Centrifuge 5430 R. This crude enzyme extract was stored at 4 °C for subsequent analysis of L-asparaginase activity [20]. The hydrolysis rate of L-asparagine was determined by measuring the liberated ammonia by the Nesslerization method [21]. One international unit (IU) of L-asparaginase is that amount of enzyme that liberates 1 μmole of ammonia in 1 min at 37 °C [22]. The enzyme was purified using a two-step process. Firstly, ammonium sulfate precipitation was employed to concentrate the protein. Subsequently, ion-exchange chromatography using diethylaminoethyl cellulose (DEAE) was performed to further purify the enzyme [20].

### Immunoblotting characterization of molecular weight and homogeneity of L-asparaginase

The lyophilized enzyme was subjected to SDS-PAGE immunoblotting to characterize the protein’s homogeneity and molecular weight. SDS-PAGE was conducted using a 10% polyacrylamide gel. The molecular masses of the protein bands were compared against the PageRuler™ Prestained Protein Ladder (cat. no. 26616, Thermo Scientific). The protein bands were stained with Coomassie brilliant blue R-250, as described in [23]. Supplementary materials show the full-length blot.

### *In vitro* anticancer activity of L-asparaginase

The cytotoxicity study was demonstrated using human breast cancer cell lines MCF-7 and MDA-MB-231 and colon cancer cell line HCT116. Cell lines were obtained from VACSERA, Egypt. The MTT assay is a colorimetric assay for measuring cells viability, based on the ability of living cells to metabolize tetrazolium salt MTT to formazan. Formazan has a purple color that is quantified colorimetrically using spectrophotometer Agilent G6860, USA, at 570nm wavelength [24].

## Results

### Collection of *Cladosporium* isolates

**Fig. 1:**
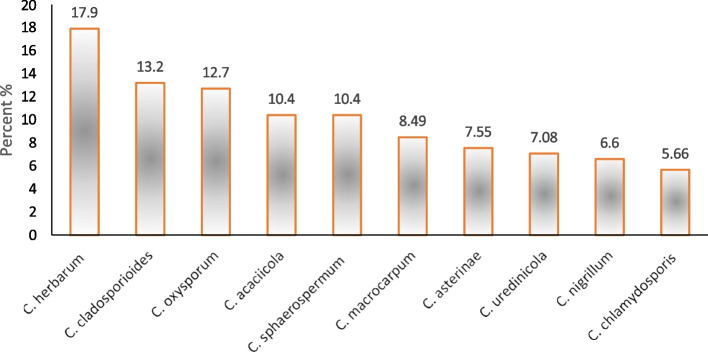
Isolation frequency of *Cladosporium* species

A total number of 886 fungal colonies were isolated from different five distinct geographical regions in Cairo Governorate. A 212 *Cladosporium* isolates from 10 different species were isolated at a 23.9% isolation rate as shown in Table 1 and Fig. 1.

**Table 1:** Frequency of *Cladosporium* isolates at different geographical regions

Region	City/town	Region code	Samples/site	*Cladosporium* isolates	*Rate of isolation (%)	**Rate of isolation (%)
East	El Salam	E1	42	15	35.71	7.075
	El Nozha	E2	54	12	22.22	5.66
	Nasr City	E3	61	9	14.75	4.245
	Ain Shams	E4	35	13	37.14	6.132
West	Bolaq	W1	37	18	48.65	8.491
	Kasr El Nile	W2	69	12	17.39	5.66
	Zamalek	W3	54	13	24.07	6.132
North	Matarya	N1	77	8	10.39	3.774
	Shobra	N2	95	17	17.89	8.019
South	Maadi	S1	37	15	40.54	7.075
	Helwan	S2	84	27	32.14	12.74
	Dar Elsalam	S3	36	9	25	4.245
DT	Mokattam	DT	205	44	21.46	20.75
Total			886	212	23.9	-

^*^Percent related to the number of isolates from each city

^**^Percent related to the total number of *Cladosporium* isolates, i.e. 212

### Identification of *Cladosporium* species

#### Macroscopical and microscopical identification

**Fig. 2:**
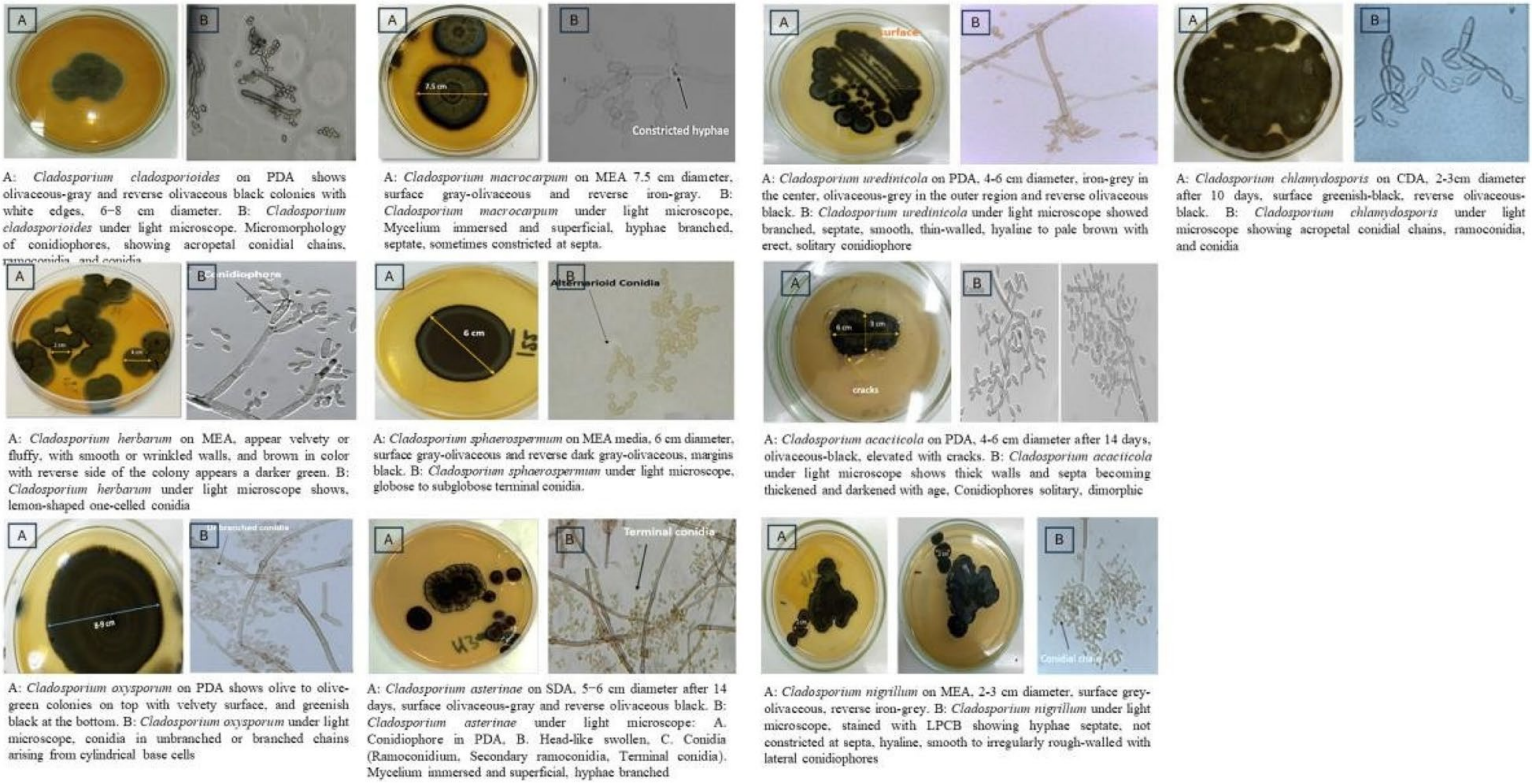
The identified *Cladosporium* species. **A** Macroscopical. **B** Microscopical identification

In the current work, macroscopical and microscopical features and biochemical properties identified a total 10 *Cladosporium* species that include *Cladosporium asterinae*, *Cladosporium uredinicola*, *Cladosporium acaciicola*, *Cladosporium macrocarpum*, *Cladosporium herbarum*, *Cladosporium nigrillum*, *Cladosporium oxysporum*, *Cladosporium cladosporioides*, *Cladosporium sphaerospermum*, and *Cladosporium chlamydosporis*. The most predominant species was *Cladosporium herbarum* (38/212) at a frequency of 17.9%, as shown in Table 2 and Fig. 2.

**Table 2:** Biochemical reactions of *Cladosporium* species

	**Casein hydrolysis test**		**Starch hydrolysis test**		**Gelatin hydrolysis test**		**Tyrosine hydrolysis test**		**Urease test**	
	No.*	**%**	No.*	**%**	No.*	**%**	No.*	**%**	No.*	**%**
***C. acaciicola***	0	0	8	36.3	0	0	19	86.36	18	81.8
***C. asterinae***	12	75	16	100	16	100	15	93.75	14	87.5
***C. chlamydosporis***	10	83.3	9	75	0	0	12	100	12	100
***C. cladosporioides***	28	100	0	0	0	0	20	71.43	19	67.9
***C. herbarum***	36	94.7	18	47.3	31	81.58	35	92.11	33	86.8
***C. macrocarpum***	0	0	0	0	8	44.44	15	83.33	17	94.4
***C. nigrillum***	12	85.7	0	0	0	0	13	92.8	0	0
***C. oxysporum***	19	70.3	0	0	24	88.89	26	96.3	0	0
***C. sphaerospermum***	22	100	0	0	20	90.91	0	0	20	90.9
***C. uredinicola***	13	86.6	9	60	0	0	12	80	10	66.7

0, negative reaction. No.*, number of positive reactions. %, positive ratio in relation to the total number of positive reaction given by each *Cladosporium* species. *C. Cladosporium*. *P*-value = 0183

### L-asparaginase production by *Cladosporium* species

#### Detection of extracellular L-asparaginase production

Among the 212 *Cladosporium* isolates tested for extracellular L-asparaginase production, 39 isolates (18.4%) were tested positive after 48 h of incubation. Six species were able to synthesize and release the enzyme, specifically *Cladosporium asterinae*, *Cladosporium cladosporioides*, *Cladosporium nigrillum*, *Cladosporium oxysporum*, *Cladosporium sphaerospermum*, and *Cladosporium uredinicola*. Screening the number of positive isolates identified *Cladosporium cladosporioides* was the predominant species, presenting 13 out of 39 strains (33.34% of the total positive strains). This was followed by *Cladosporium oxysporum*, which yielded positive tests in eight strains (20.51% of the total positive strains). Conversely, *Cladosporium sphaerospermum* and *Cladosporium asterinae* exhibited the least positive tests, with three and four strains presented (7.69% and 10.25%, respectively), as shown in Table 3.

**Table 3:** L-asparaginase producing *Cladosporium* species

*Cladosporium* species	Mean diameter (mm) after 48 h	Total number/species	*%
*C. cladosporioides* HMA-132	18 ± 0.34		
*C. cladosporioides* HMA-14	24 ± 0.97		
*C. cladosporioides* HMA-145	16 ± 0.828		
*C. cladosporioides* HMA-172	19 ± 1.883		
*C. cladosporioides* HMA-221	29.5 ± 1.5		
*C. cladosporioides* HMA-232	24.5 ± 0.5		
*C. cladosporioides* HMA-285	36 ± 1.5	13	33.33%
*C. cladosporioides* HMA-289	26 ± 1.054		
*C. cladosporioides* HMA-311	23 ± 0.752		
*C. cladosporioides* HMA-407	25 ± 0.5		
*C. cladosporioides* HMA-67	13 ± 0.23		
*C. cladosporioides* HMA-713	17 ± 0.664		
*C. cladosporioides* HMA-E3	34.5 ± 1.5		
*C. oxysporum* HMA-10	28.5 ± 1.0		
*C. oxysporum* HMA-M2	26.3 ± 1.5		
*C. oxysporum* HMA-245	28.3 ± 1.8		
*C. oxysporum* HMA-91	33 ± 1.226		
		8	20.51%
*C. oxysporum* HMA-106	46 ± 0.528		
*C. oxysporum* HMA-312	38 ± 0.2517		
*C. oxysporum* HMA-680	14 ± 0.025		
*C. oxysporum* HMA-661	22.33 ± 1.66		
*C. uredinicola* HMA-59	18 ± 1.25		
*C. uredinicola* HMA-435	14.5 ± 1.041		
*C. uredinicola* HMA-216	25.5 ± 1.88		
		6	15.38%
*C. uredinicola* HMA-712	38.33 ± 1.11		
*C. uredinicola* HMA-771	21.66 ± 2.466		
*C. uredinicola* HMA-108	26 ± 1.987		
*C. nigrillum* HMA-265	26 ± 0.62		
*C. nigrillum* HMA-62	21 ± 0.41		
*C. nigrillum* HMA-192	17 ± 0.775	5	12.82%
*C. nigrillum* HMA-388	15 ± 0.515		
*C. nigrillum* HMA-455	22.33 ± 1.258		
*C. sphaerospermum* HMA-182	22 ± 1.84		
*C. sphaerospermum* HMA-268	18 ± 1.0	3	7.69%
*C. sphaerospermum* HMA-316	37.17 ± 2.255		
*C. asterinae* HMA-84	23 ± 1.51		
*C. asterinae* HMA-173	21.17 ± 2.75		
		4	10.25%
*C. asterinae* HMA-188	17 ± 1.5		
*C. asterinae* HMA-728	28 ± 1.0		

Data is presented as mean ± standard deviation (SD). The pH of the medium is 6.2. *C.:Cladosporium*. *%, percentages were correlated to the total number of positive test, i.e. *n* = 39

#### Determination of extracellular L-asparaginase activity

**Fig. 3:**
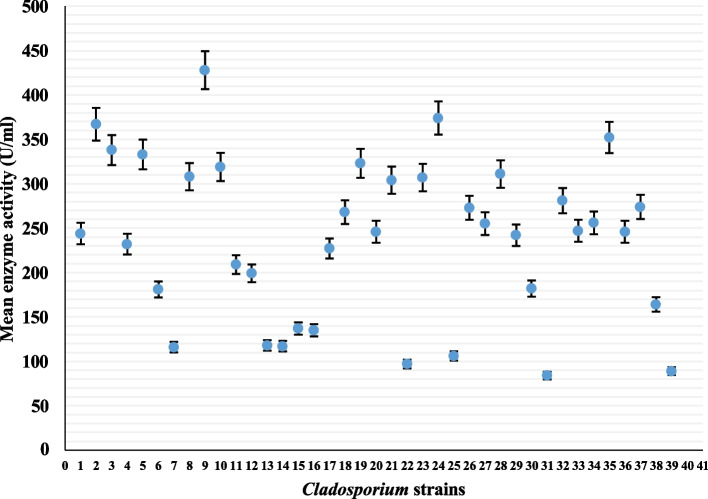
Determination of L-asparaginase enzyme activity recovered from *Cladosporium* species. A 39 *Cladosporium* isolates expressing the highest L-asparaginase activity were selected from the total isolates

*Cladosporium cladosporioides* HMA-221 was found to have the highest extracellular L-asparaginase enzyme activity among all tested *Cladosporium* strains. The enzyme’s activity ranged from 84 to 428 U/ml and was determined using a standard curve generated from different concentrations of ammonium chloride. One international unit (IU) of microbial L-asparaginase enzyme is defined as the amount of enzyme capable of producing 1 µmol of NH_3_ per min at 37 °C (Table 4 and Fig. 3).

**Table 4:** Absorbance of ammonium chloride (NH_4_Cl) at different concentrations

NH_4_Cl µmole/ml	Absorbance	Absorbance^a^
500	0.766	0.6696
400	0.622	0.5256
300	0.548	0.4516
200	0.388	0.2916
100	0.264	0.1676
50	0.155	0.0586
0	0.0964	0

^a^Absorbance after subtraction from control (blank is saline)

#### Quantitation of L-asparaginase produced from *Cladosporiumcladosporioides* HMA 221

The *Cladosporium cladosporioides* HMA-221 strain, expressing L-asparaginase with the highest enzymatic activity, was utilized. The protein content was determined using a standard curve equation derived from Lowry’s method. The purification process of the extracellular L-asparaginase from the *Cladosporium cladosporioides* HMA-221 strain involved three steps: ammonium sulfate precipitation, gel filtration, and ion-exchange chromatography. The enzyme was firstly isolated by saturating it with ammonium sulfate. It was then fractionated at 65% saturation and dialysis, resulting in a significant increase in the specific activity of L-asparaginase from 5.67 to 14.38 IU/mg, with a 2.53-fold purification. The dialyzed ammonium sulfate precipitate was subsequently passed through a Sephadex G100 gel filtration column, eluted with 0.05-M Tris–HCl (pH 8.4) buffer. This process separated the enzyme into one major peak and three minor peaks. The protein signal in each fraction was quantified by measuring the optical density at 660 nm. Fractions showing a single peak (F18–23) with notable L-asparaginase activity were pooled for further dialysis, resulting in a decrease in total protein content from 665.55 to 148.62 mg. The specific activity of L-asparaginase then increased to 169.32 U/mg, achieving a purification efficiency of 72.73% and a 29.83-fold increase post-gel filtration chromatography. The protein, which was initially lyophilized and purified through gel filtration chromatography, was processed through additional purification via CM-Sephadex C50 ion-exchange chromatography. The elution profile showed that the enzyme was eluted in six fractions (F8–F13) as a single peak. Those fractions were combined, dialyzed, and then lyophilized. The purified L-asparaginase had a specific activity of 370.34 U/mg and a yield of 60.77%.

#### Determination of molecular weight and homogeneity by SDS-PAGE

**Fig. 4:**
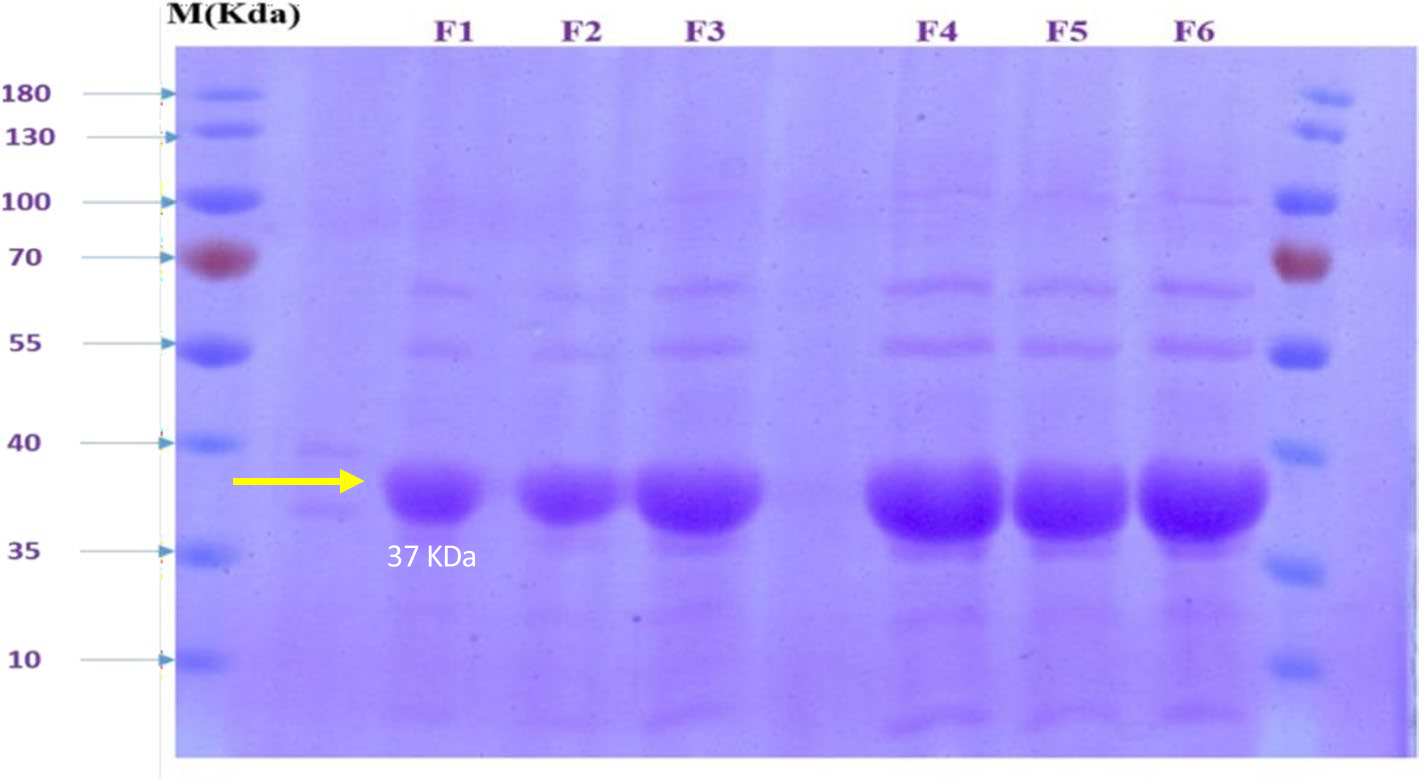
SDS-PAGE analysis of L-asparaginase produced from *C. cladosporioides* HMA 221 following chromatographic purification. From left to right: Lane M, molecular weight markers; lanes F1–F8 are purified protein fractions on CM-Sephadex C50 ion exchange chromatography

The CM-Sephadex C-50 chromatographic purification of L-asparaginase showed major peak consisted of 6 fractions (F8-F13) presenting a single protein band with a single peak of enzyme activity observed in gel filtration elution profile. It indicated that only one distinctive protein band for the pure preparation of L-asparaginase with an apparent molecular weight of 35–40 kDa protein was obtained via comparing the migration distances of standard marker proteins (BIORAD medium range of 15 to 200 kDa) as illustrated in Fig. 4.

### Anticancer activity of *Cladosporiumcladosporioides* HMA-285 L-asparaginase in vitro

**Fig. 5:**
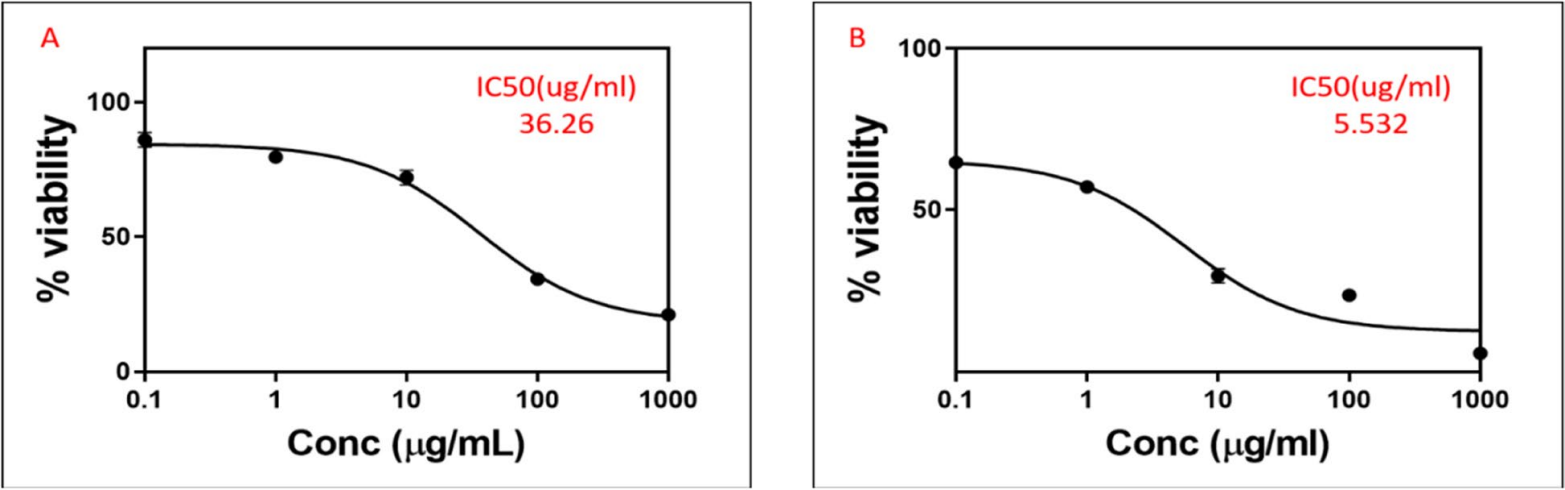
Dose–response curves of *C. cladosporioides* L-asparaginase against MCF 7 breast cancer cell line (**A**) and doxorubicin as control (**B**)

Two distinct breast cancer cell lines, MCF-7 and MDA-MB-231, were subjected to various concentrations of *Cladosporium cladosporioides* HMA-221 L-asparaginase (ranging from 0.1 to 1000 μg) to evaluate its anticancer potential. *Cladosporium cladosporioides* L-asparaginase-induced gradient toxicity agonist malignant breast cells. The viability of MCF-7 cells decreased in a fashion that correlated with the escalating concentration gradient of L-asparaginase, reaching its nadir at 1000 μg/ml in comparison to doxorubicin, which served as the control. Furthermore, the IC_50_ was significantly higher for L-asparaginase compared to doxorubicin, with values of 36.26 and 5.532, respectively, as shown in Fig. 5.

**Fig. 6:**
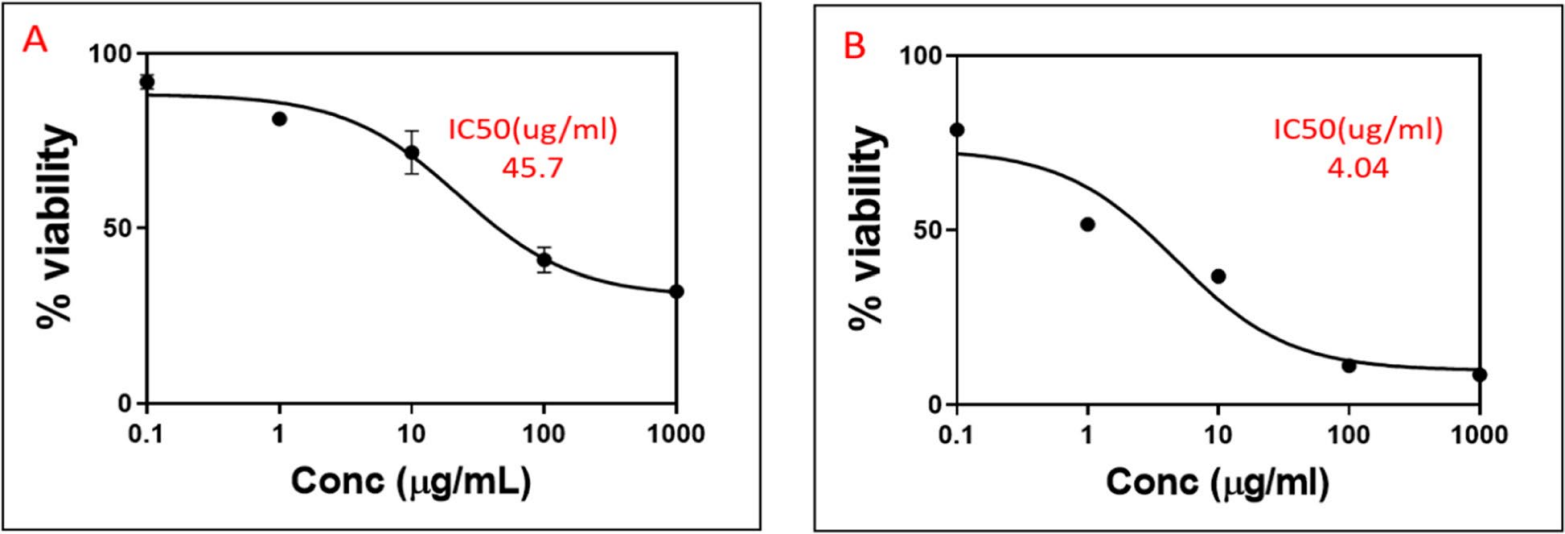
Dose–response curves of *C. cladosporioides* L-asparaginase against MDA 231 breast cancer cell line (**A**) and doxorubicin as a control (**B**)

The viability (%) of MDA-231 cells decreased with the escalating concentrations of L-asparaginase, reaching its lowest value at an enzyme concentration of 1000 μg/ml, in contrast to doxorubicin. Furthermore, the IC50 values were considerably higher for L-asparaginase compared to doxorubicin, with respective values of 45.7 and 4.04, as illustrated in Fig. 6.

**Fig. 7:**
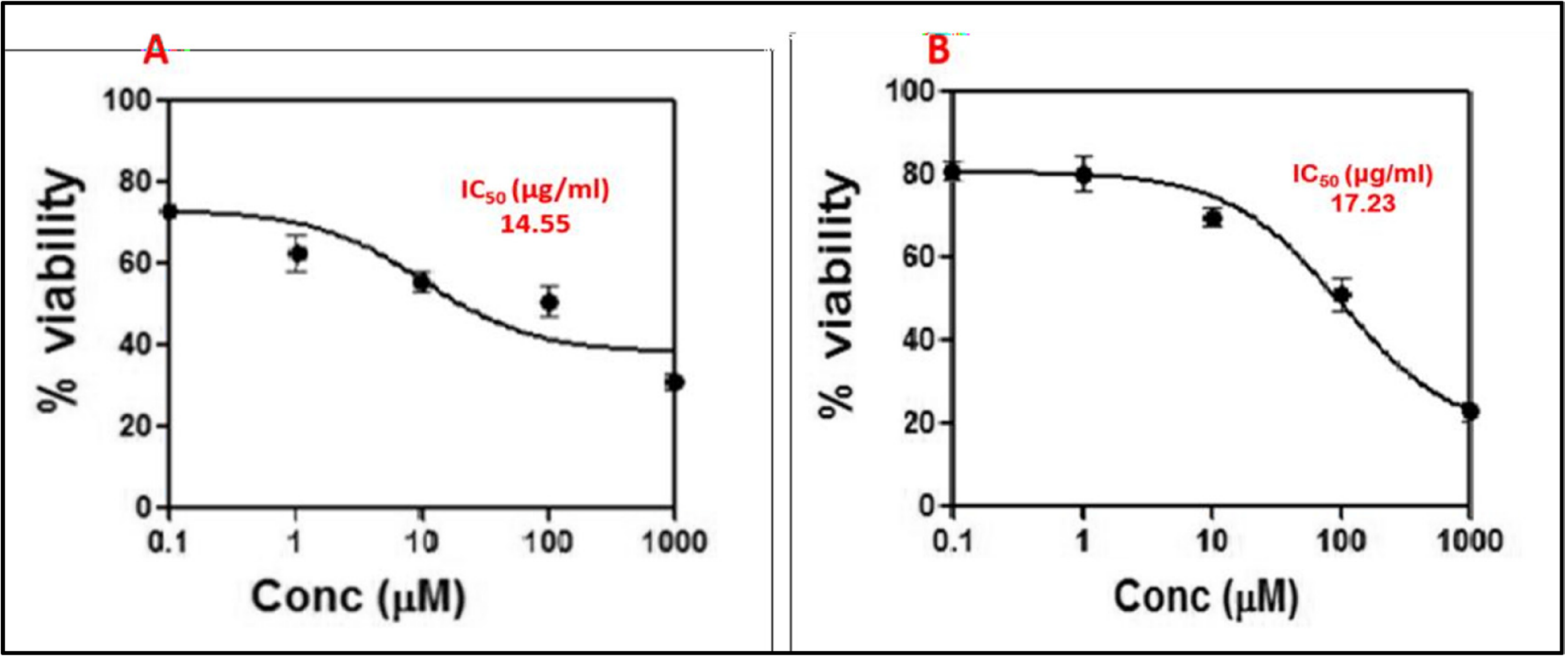
Dose–response curves of *C. cladosporioides* L-asparaginase against HCT 116 colon cancer cell line (**A**) and doxorubicin as a control (**B**)

The viability (%) of HCT 116 cell lines was used as an indicator of cell toxicity after cells were treated with different concentrations of L-asparaginase produced by *Cladosporium cladosporioides* HMA 221 for 72 h. The viability of HCT 116 colon cancer cells decreased proportionally with the increase of L-asparaginase enzyme concentration, reaching the least value at 1000 μg/ml, in comparison with doxorubicin as a control. In addition, the IC50 was markedly higher with L-asparaginase than doxorubicin, with values 14.55 and 17.23 respectively, as shown in Fig. 7.

## Discussion

The present study demonstrated the production of L-asparaginase enzyme by *Cladosporium* species. Upon screening of *Cladosporium* species for production of the antiproliferative L-asparaginase enzyme, 39 isolates (18.4%) showed L-asparaginase production activity with pink zone diameter. Among the tested *Cladosporium* species that actively expressed the L-asparaginase enzyme, six species were able to synthesize and release the enzyme: *Cladosporium cladosporioides*, *Cladosporium sphaerospermum*, *Cladosporium oxysporum*, *Cladosporium nigrillum*, *Cladosporium asterinae*, and *Cladosporium uredinicola*. The most L-asparaginase enzyme producing isolate yielded enzyme activity of 167 IU/ml, followed by 149 IU/ml, and both isolates were identified as *Cladosporium acaciicola*. This data was comparable with that of Kumar et al. (2013) [22] who reported 10 positive L-asparaginase *Cladosporium* isolates from a total 50 *Cladosporium* isolates (20%). Another study by Sanjotha and Manawadi [25] showed much lower results where the *Cladosporium* isolates expressing L-asparaginase presented 8.64% from the total isolates. Those observations were similar to the study of Nnanna et al. [26], who reported positive L-asparaginase from *Cladosporium* fungi; mostly, *Cladosporium tenuissimum* was the predominant species. Furthermore, El-Hadi et al. (2019) [27] reported similar results of L-asparaginase expression from *Streptomyces* *spp*. In contrast, higher isolation rate was reported in the study of Alrumman et al. (2019) [28], in which 28 marine bacteria species expressing L-asparaginase out of 40 isolates (70%) were identified. In the current study, toxicity of L-asparaginase expressed by *Cladosporium cladosporioides* HMA-221 against breast cancer cell lines MCF-7 and MDA-MB-231, and colon cancer cell line HCT 116, was assessed. In vitro cultures were subjected to different concentrations of *Cladosporium cladosporioides* HMA-221. The present work agrees with the study of Benchamin et al. (2019) [29] on the fungus *Aspergillus fumigatus* L-asparaginase’s antiproliferative effect on MDA-MB-231 cell line. The 71%, 87.7%, and 96.5% of cell death occurred when 5 U, 10 U, and 20 U of drugs were applied respectively. Those findings were comparable to other studies [30, 31] where L-asparaginase from marine *Bacillus* species and *Bacillus licheniformis* strains exhibited the highest cytotoxic effect against cancer cell lines Jurkat clone E6-1, MCF-7, and K-562, with IC_50_ values of 0.22, 0.78, and 0.153 IU, respectively. However, the results of the current study were superior to those described previously by Farag et al. (2015) [16] for *Halomonas* *elongate*, where L-asparaginase inhibited the growth of human leukemia cell lines with IC_50_ values of 1–2 U/mL. Furthermore, L-asparaginase from *Bacillus spp*. R36 inhibited the growth of two human cell lines (HCT-116 and HepG-2), with IC_50_ values of 218.5 and 112.19 μg/mL, respectively [32]. In addition, Shafei et al. (2015) [33] described L-asparaginase that inhibited the growth of three human cell lines, with IC_50_ values of 14, 12.5 and 37 μg/mL against breast, hepatocellular and prostate carcinoma, respectively. The aforementioned data reconceal with the roles of L-asparaginase that has been shown to inhibit the glycosylation of several forms of newly synthesized proteins [34]. El-Naggar et al. (2016) [35] suggested that L-asparaginase can alter the interactions between the microvasculature of endothelial cells, colon cancer cells, and extracellular matrix components, resulting in colon cancer cell disruption. After being converted into oxaloacetic acid, asparagine is involved in the Krebs cycle, influencing cell metabolism [36].

## Significance and conclusion

L-asparaginase, a commercially predominant enzyme, accounts for approximately 40% of the global enzyme sales. It has particularly gained prominence in the medical field for its efficacy in treating acute lymphoblastic leukemia (ALL). Our findings demonstrate that *Cladosporium* species can efficiently express and secrete L-asparaginase with cytotoxic activity against breast and colon cancer cell lines. None-of-the-less, this suggests its in vitro potential for pharmaceutical applications. This article describes molecular approaches to further assess the hypothesis that the ectopic expression of L-asparaginase may increase the susceptibility of tumor cells to cytotoxic drugs that target apoptosis/survival and cell migration/invasion cascades. Further, enhancing the sensitivity of tumor cells to anticancer agents and potentially protecting normal cells from the chemo-radio cytotoxic effects, L-asparaginase, could offer a novel approach for cancer therapy.

### Future research

Different models have explored the implication of L-asparaginase in types of cell death: apoptosis, autophagy, and necrosis. To further investigate the impact of L-asparaginase on cancer cell survival and death, we propose to study the impact of L-asparaginase ectopic expression on the susceptibility of breast and colon tumor cells to cell death in vitro. We propose generating and testing a lentiviral system to transduce the L-asparaginase gene into cancer cells and assess their viability in vitro. Cell survival can be measured using cell viability assays in a high-throughput context, i.e., ATPlite assays. We aim to validate in primary human cells the stable ectopic expression of L-asparaginase in Lentivirus that effectively modulate L-asparaginase across different eukaryotic species by selecting cell populations with stable upregulated L-asparaginase transcripts. Once we identify signaling cascades, response markers identified in vitro will be examined for their expression in breast tumor blocks and the correlation between their expression levels and tumorigenicity.

### Limitations

We were limited by the available resources for conducting a histopathological analysis of the L-asparaginase expression in the studied tissues. Our extended current research will take such valuable comments into account.

## Supplementary Information

Supplementary Material 1. Full-length blot.

## Data Availability

No datasets were generated or analysed during the current study.
