# Supplementary material for: Cytotoxicity of L-asparaginase from eucaryotic Cladosporium species against breast and colon cancer in vitro
Source: J Egypt Natl Canc Inst. 2025 May 3;37:33. doi: 10.1186/s43046-025-00270-6 (PMC13313450; doi:10.1186/s43046-025-00270-6)

CYTOTOXICITY OF L- ASPARAGINASE FROM EUCARYOTIC CLADOSPORIUM SPECIES AGAINST BREAST AND COLON CANCER IN VITRO


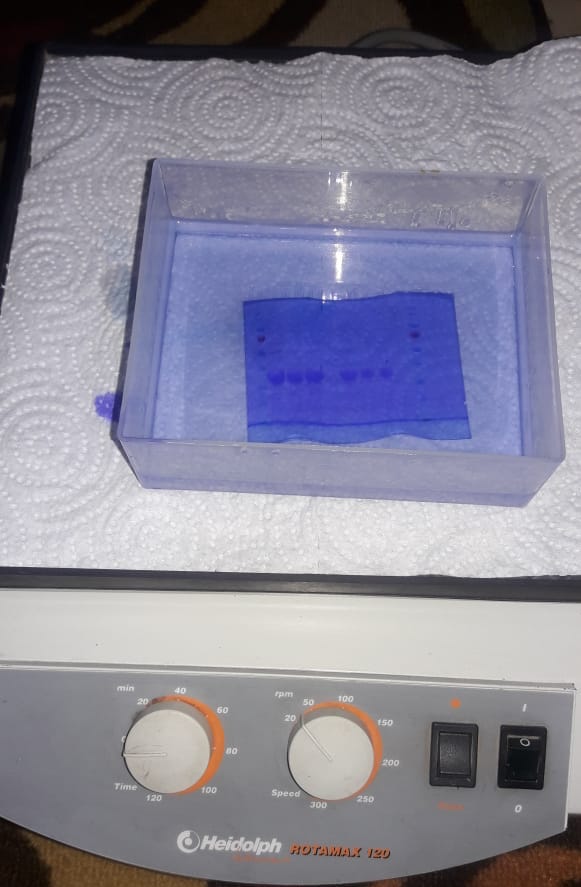


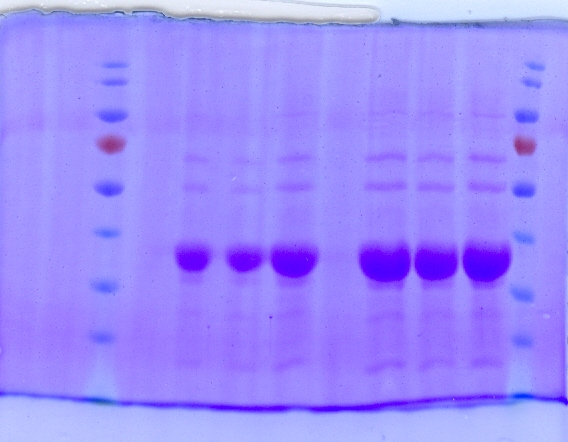


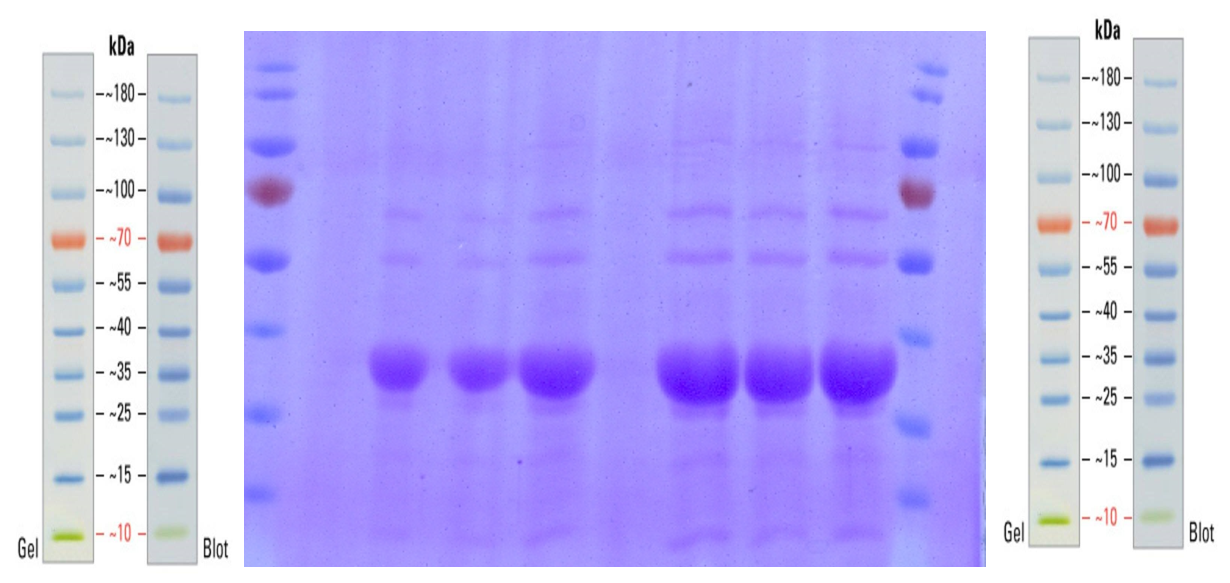

Supplement: Supplementary file 1 — Supplementary Material 1. Full-length blot. [file 43046_2025_270_MOESM1_ESM.docx]
